# Coronary vessel wall thickening in asymptomatic young HIV-infected patients vs. controls using phase-sensitive dual inversion recovery (PS-DIR) MRI

**DOI:** 10.1186/1532-429X-15-S1-P223

**Published:** 2013-01-30

**Authors:** Khaled Z Abd-Elmoniem, Dima Hammoud, Julia Purdy, Rohan Hazra, Roderic I Pettigrew, Colleen Hadigan, Ahmed Gharib

**Affiliations:** 1 Biomedical and Metabolic Imaging Branch, NIDDK/NIH, Bethesda, MD, USA; 2 Center for Infectious Disease Imaging, Clinical Center, NIH, Bethesda, MD, USA; 3 Critical Medicine Department, Clinical Center, NIH, Bethesda, MD, USA; 4 National Institute of Allergy and Infectious Diseases, NIH, Bethesda, MD, USA; 5 National Institute of Child Health and Human Development, NIH, Bethesda, MD, USA; 6 National Cancer Institute, NIH, Bethesda, MD, USA

## Background

HIV-infected patients demonstrate premature and more severe vascular disease relative to controls. Therefore, development of a reproducible non-invasive vascular imaging biomarker in this population is important. The purpose of this study was to assess coronary artery wall thickness in young HIV-infected patients who acquired HIV in early life compared to healthy controls using a novel black-blood coronary vessel wall MR imaging technique.

## Methods

We prospectively studied 20 young HIV-infected adults and 12 HIV-uninfected control subjects. Participants were free of known active cardiovascular disease or symptoms at the time of the scan. MR imaging of the proximal right coronary artery (RCA) vessel wall was performed using phase-sensitive dual inversion-recovery (PS-DIR) black-blood vessel wall imaging. Group comparisons were performed to evaluate differences in proximal RCA wall thickness and potential associations between clinical variables and wall thickness were evaluated using linear regression analyses.

## Results

HIV-infected subjects ranged in age from 15-29 years, with an average age of 21.1 years (50% male). HIV-uninfected subjects ranged in age from 23 to 47 years, with an average age of 29.3 years (42% male). The average RCA wall was significantly thicker in HIV-infected patients compared to controls (p-value= 0.0015), despite the fact that controls were significantly older (p=0.0001). In HIV-infected subjects, linear regression analysis did not show significant correlation between the RCA wall thickness and duration of disease, current or nadir CD4 counts, levels of total cholesterol, LDL cholesterol or triglycerides.

## Conclusions

We have demonstrated statistically significant increased proximal RCA wall thickness as a likely surrogate marker of generalized vascular disease in a group of young patients who acquired HIV in early life compared to HIV-uninfected subjects. Although we did not find a significant correlation between the vessel wall thickness and markers of infection such as CD4 counts, we believe studying a larger sample size is warranted to determine more definitively if such a correlation exist.

## Funding

This work has been funded by the National Institutes of Health

**Figure 1:**
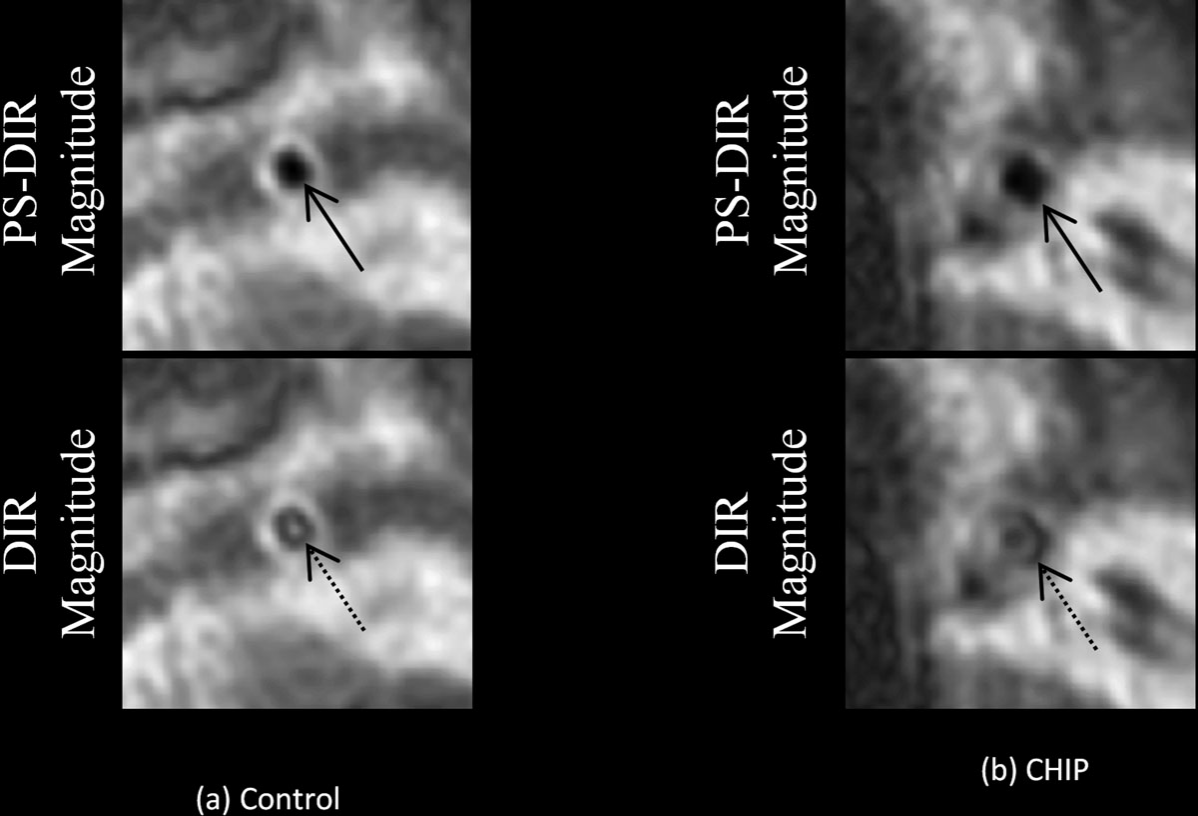


**Figure 2:**
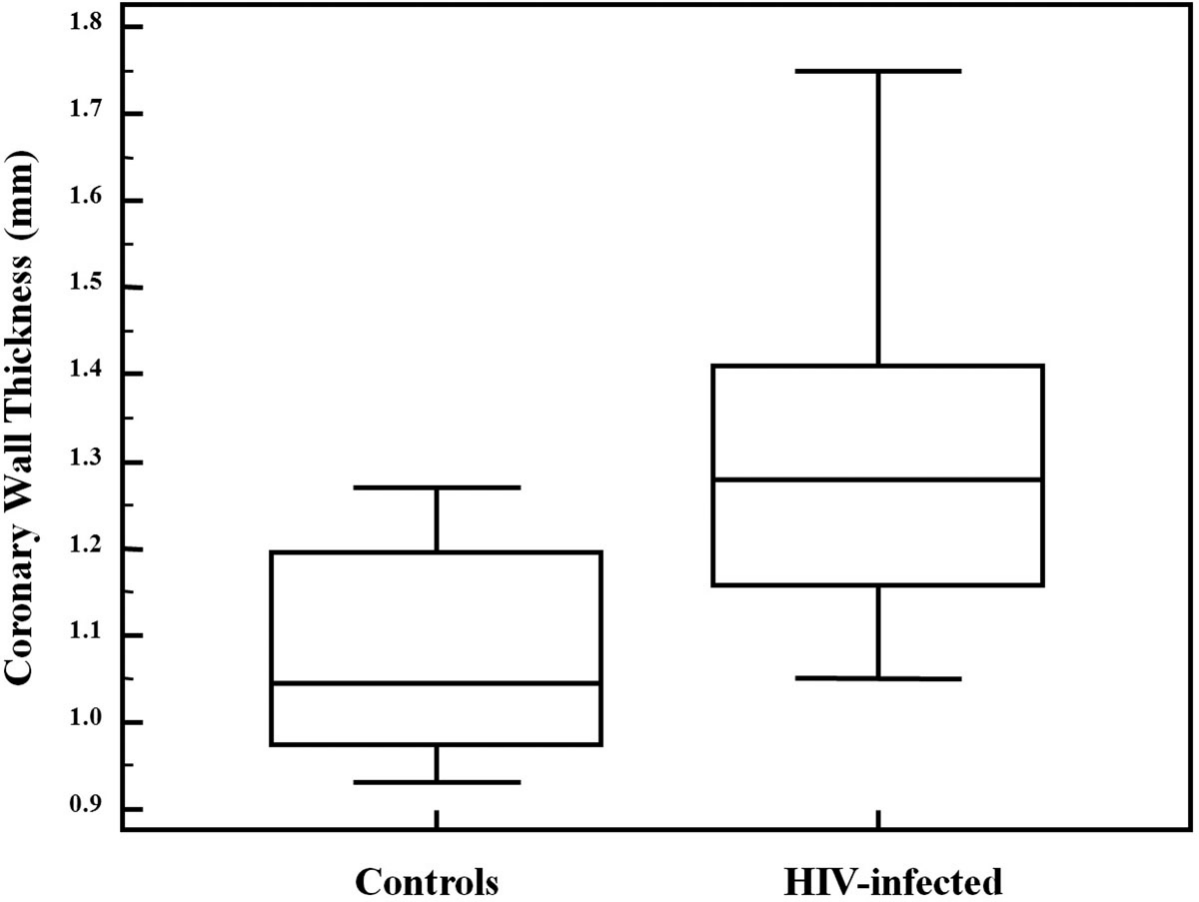
Despite older age of controls, RCA vessel wall thickness was increased in HIV vs. Control. HIV: 1.31±0.21 mm Control: 1.07±0.12 mm

